# Longitudinal patterns of fluid overload, blood volume and vascular refilling: a prospective study in patients on maintenance hemodialysis

**DOI:** 10.1093/ckj/sfaf199

**Published:** 2025-06-27

**Authors:** Sebastian Mussnig, Simon Krenn, Max Waller, Michael Schmiedecker, Amelie Kurnikowski, Janosch Niknam Saeidi, Luis Naar, Christopher C Mayer, David Keane, Daniel Schneditz, Manfred Hecking, Leszek Pstras

**Affiliations:** Medical University of Vienna, Center for Public Health, Department of Epidemiology, Vienna, Austria; Medical University of Vienna, Department of Medicine III, Division for Nephrology and Dialysis, Vienna, Austria; Medical University of Vienna, Center for Public Health, Department of Epidemiology, Vienna, Austria; Medical University of Vienna, Department of Medicine III, Division for Nephrology and Dialysis, Vienna, Austria; AIT Austrian Institute of Technology GmbH, Center for Health & Bioresources, Medical Signal Analysis, Vienna, Austria; Medical University of Vienna, Center for Public Health, Department of Epidemiology, Vienna, Austria; Medical University of Vienna, Department of Medicine III, Division for Nephrology and Dialysis, Vienna, Austria; Clinic Favoriten, Department of Medicine I with Nephrology, Intensive Medicine, Psychosomatics and Diabetology, Vienna, Austria; Medical University of Vienna, Department of Medicine III, Division for Nephrology and Dialysis, Vienna, Austria; Clinic Ottakring, Vienna, Austria; Medical University of Vienna, Center for Public Health, Department of Epidemiology, Vienna, Austria; Medical University of Vienna, Department of Medicine III, Division for Nephrology and Dialysis, Vienna, Austria; Medical University of Vienna, Center for Public Health, Department of Epidemiology, Vienna, Austria; Clinic Landstrasse, 1^st^ Department of Medicine, Vienna, Austria; Medical University of Vienna, Department of Medicine III, Division for Nephrology and Dialysis, Vienna, Austria; AIT Austrian Institute of Technology GmbH, Center for Health & Bioresources, Medical Signal Analysis, Vienna, Austria; University of Galway, CURAM, Center for Research in Medical Devices, Galway, Ireland; Medical University of Graz, Division of Physiology & Pathophysiology, Otto Loewi Research Center for Vascular Biology, Immunology and Inflammation, Graz, Austria; Medical University of Vienna, Center for Public Health, Department of Epidemiology, Vienna, Austria; Medical University of Vienna, Department of Medicine III, Division for Nephrology and Dialysis, Vienna, Austria; Kuratorium for Dialysis and Transplantation (KfH), Germany; Nalecz Institute of Biocybernetics and Biomedical Engineering, Polish Academy of Sciences, Warsaw, Poland

**Keywords:** bioimpedance spectroscopy, blood volume, fluid overload, hemodialysis, vascular refilling

## Abstract

**Introduction:**

Patients on maintenance hemodialysis accumulate excess fluid between treatments. Intradialytic removal of fluid via ultrafiltration is partly compensated by vascular refilling from the interstitial space. Associations between whole-body fluid status and blood volume were previously investigated on the population level. The aim of this observational cohort study was to assess longitudinal changes in fluid compartment volumes on an intra-patient level.

**Methodology:**

Pre-dialysis bioimpedance spectroscopy measurements and absolute blood volume estimations were conducted in maintenance hemodialysis patients during 14 consecutive dialysis treatments over 5 weeks. Blood volume was determined using the dialysate bolus method. Longitudinal changes were evaluated using linear mixed models. Correlations were analyzed with repeated measures correlation coefficients (${\rho _{rm}}$).

**Results:**

Twenty-five patients were included in the final analysis [88% male, median (quartile 1, quartile 3) age and dialysis vintage of 66.0 years (48.0, 74.0) and 23.5 months (13.5, 34.5), respectively]. Pre-dialysis fluid overload significantly decreased from the first to the third treatment within the week (*β* = −0.38, *P* < .01) with no significant within-week changes in euvolemic body mass (*β*= −0.04, *P* = .78) or absolute blood volume at treatment start (*β* = −0.06, *P* = .65). Fluid overload did not correlate with absolute (${\rho _{rm}}$ = 0.10, *P* = .65) or specific blood volume (${\rho _{rm}}$=0.06, *P* = .78) at treatment start on an intra-patient level, but correlated moderately with refilling volume (${\rho _{rm}}$ = 0.46, *P* < .01).

**Conclusions:**

The observed lack of intra-patient correlations between pre-dialysis fluid overload and blood volume suggests that excess fluid may not necessarily accumulate proportionally in the interstitial and intravascular space, thus challenging previous assumptions regarding within-week changes in fluid compartments.

KEY LEARNING POINTS **What was known:**

- Fluid overload decreases over the thrice-weekly hemodialysis cycle.
- Previous studies have reported moderate correlations between pre-dialysis fluid overload and blood volume on the population level.

**This study adds:**

- In patients on intermittent maintenance hemodialysis, pre-dialysis blood volume, and intracellular fluid volume did not show a decreasing trend over the weekly cycle, as seen with fluid overload and extracellular fluid volume.
- Fluid overload and blood volume did not correlate on an intra-patient level.

**Potential impact:**

- Excess fluid may accumulate predominantly in the interstitial space in patients on maintenance hemodialysis, challenging previous assumptions.

## INTRODUCTION

One of the main aims of hemodialysis is to restore normal fluid status in patients suffering from kidney failure requiring kidney replacement therapy. Adequate fluid status is achieved by removing excess fluid via ultrafiltration of blood plasma [1], which typically results in a decrease of blood volume throughout the treatment [2]. ‘Refilling’ of the vascular bed with fluid from the interstitial space partially compensates for the removal of fluid from blood in the dialyzer [3]. The magnitude of transcapillary vascular refilling during dialysis depends on microvascular hydraulic permeability [3] and the imbalance between Starling forces [4], i.e. the differences in osmotic and hydrostatic/hydraulic pressures in the intra- and extravascular compartments [4], which vary throughout the treatment [5].

The interdialytic change of body mass is a common surrogate for fluid overload, assuming that gains in body mass between treatments are exclusively due to excess fluid [1]. Bioimpedance spectroscopy allows the quantification of body fluid volumes by measuring tissue responses to weak alternating electrical currents of various frequency [6]. Through empirically derived models, the measured impedances serve to estimate volumes of the extracellular and intracellular fluid [7], and fluid overload, as well as lean and fat tissue mass [8]. Based on the estimated fluid overload, “euvolemic” body mass is then the body mass without fluid overload, which may help in setting ultrafiltration goals [8].

While body mass changes and bioimpedance-spectroscopy-based measurements approximate whole-body fluid status, excess fluid needs to be ultrafiltered from blood plasma. Many modern dialysis machines are equipped with non-invasive sensors which continuously measure intradialytic relative changes in blood volume [9]. The course of these relative changes is a direct effect of ultrafiltration and vascular refilling [10]. Absolute blood volume can be estimated from the response of relative blood volume to a fluid infusion of known volume, akin to classic indicator dilution methods [11–14]. Knowledge of absolute blood volume and ultrafiltration volume throughout the treatment may then inform on refilling volume, rate, and fraction at any given time during dialysis [15, 16].

Associations between fluid overload, blood volume, and vascular refilling measures were previously explored in multiple population-level, cross-sectional analyses [15–17]. However, fluid status undergoes constant changes in patients on maintenance hemodialysis [18, 19] due to the intermittent nature of fluid removal by ultrafiltration and different levels of fluid accumulation over short and long treatment intervals in the typical thrice-weekly treatment schedule. The question therefore arises as to what degree pre-dialysis fluid status, in particular blood volume assessed on a given day is representative of the whole week. The aim of this study was to explore the variability of fluid volumes and the association between fluid overload with a recently introduced measure of absolute blood volume on the intra-patient level over multiple consecutive hemodialysis treatments covering repeated weekly treatment cycles.

## MATERIALS AND METHODS

### Population and ethics

This study was an exploratory, hypothesis-generating analysis of the dataset collected within the project “Closing the Loop in Hemodialysis: A Precision Medicine Approach—Part B (Establishing an Exploratory Dialysis Data-Pool)”, the results of which were previously published elsewhere [20, 21]. Adult non-pregnant patients receiving regular thrice-weekly maintenance hemodialysis were recruited at the chronic hemodialysis ward of Vienna General Hospital (Vienna, Austria) to participate in longitudinal bioimpedance spectroscopy measurements and absolute blood volume estimations. Written informed consent was obtained from all participants. The study was approved by the ethics committee of the Medical University of Vienna (committee vote number 2057/2020) and adhered to the Declaration of Helsinki.

### Dialysis treatment

Measurements were conducted over 14 consecutive dialysis treatments in five consecutive weeks (with regular thrice-weekly treatments), starting at the second dialysis treatment in the first week and ending with the third dialysis treatment in the fifth week (31 days in total).

For the duration of the study, all patients were switched to hemodiafiltration treatment in pre-dilution mode to allow for online bolus infusion. Nurses were instructed to discard the priming saline. Patients were initially treated with either Fresenius 5008 (Fresenius Medical Care, Bad Homburg, Germany) or DBB-EXA (Nikkiso Co., Ltd, Tokyo, Japan) dialysis machines. Following the third week, all patients were switched to the alternative machine model. Dialysate and ultrafiltration were prescribed at the discretion of the treating physician throughout the study.

### Data collection

#### Body composition

Extracellular and intracellular fluid volumes and fluid overload were estimated before each of the 14 treatments using the Body Composition Monitor (software version 3.2.5, Fresenius Medical Care) in supine wrist-to-ankle electrode configuration.

#### Blood volume

Absolute blood volume at treatment start was estimated during dialysis treatments 3–14 as described elsewhere [11]. Approximately 60 min into the treatment 240 ml of ultra-pure dialysate were infused in pre-dilution mode at an infusion rate of 200 ml/min. The infusion volume was added to the target ultrafiltration volume at treatment start to achieve the desired net ultrafiltration. Relative blood volume was continuously recorded to the electronic hospital data system at sampling rates of 3/min and 1/min for DBB-EXA and 5008 dialysis monitors, respectively.

#### Blood pressure

Peripheral blood pressure was measured oscillometrically with the blood pressure cuff of the dialysis machine before dialysis, after dialysis and every 30 to 60 min during dialysis depending on patient preferences. Individual treatments were classified as being complicated by intradialytic hypotension if any intradialytic blood pressure measurement fulfilled the Nadir90/100 criteria [22].

### Estimation of absolute blood volume

The quantification of the infusion-induced increase in relative blood volume required for the calculation of absolute blood volume (Equation (1) in Supplemental Methods 1) was performed using a previously described algorithm [14]. The algorithm was slightly modified to account for differences in data characteristics from DBB-EXA measurements compared with those from 5008. In particular, data from DBB-EXA had a higher sampling rate, which on the one hand made the analysis possible without data interpolation, but on the other hand required removing occasional spikes in the blood volume signals and ignoring the immediate post-infusion period of data showing in some cases an apparent large increase (or overshoot) in relative blood volume. All estimates were manually screened for implausible results, which were excluded where necessary.

### Time course of blood volume and vascular refilling

Relative blood volume and cumulative ultrafiltration volume were median-filtered per treatment with a time window of 313 seconds. Time-dependent absolute and specific blood volumes were subsequently computed according to Equations (2) and (3) listed in the Supplemental Methods 1. Volume and rate of vascular refilling as well as the fraction of ultrafiltration compensated by vascular refilling (refilling fraction, both of rates and volumes) were calculated according to Equations (4–8) in the Supplemental Methods 1. Ultrafiltration rates, refilling rates and fractions at start and end of the treatment were averaged over the first and last 30 min of the treatment, respectively.

### Statistical analyses

Continuous variables were summarized as medians (quartile 1, quartile 3). Serial data collected throughout the study were visualized using line plots and as mean values per day and per treatment within the week, including bootstrapped 95% confidence intervals of the mean. The associations of bioimpedance-derived and blood-volume-derived variables, and ultrafiltration volume and rate with study day (day 1 to day 31) and treatment within the week (first, second, and third) were analyzed using linear mixed models. Fixed effects included study day, treatment within the week, and the confounders age and sex, all modeled together. Random intercepts were included for each patient, along with random slopes for study day. The Akaike information criterion was calculated for each model, both with and without random slopes, and the model with the lowest score was selected for reporting. The missingness of ultrafiltration volumes and rates was assumed to be completely at random, while the missingness of bioimpedance- and blood-volume-derived variables was assumed to be at random. *Post hoc* power analyses are described in Supplemental Methods 2. Coefficients of variation were calculated for each analyzed variable per patient based on data from the whole study and were subsequently averaged over the entire cohort. Intra-patient correlations between the variables were investigated via repeated measures correlation coefficients (${\rho _{rm}}$, [23]). Cohort-averaged time-dependent estimates of blood volume and vascular refilling measures were visualized by fitting no-linear smooth functions to data from all patients and all treatments (method “gam”). *P* values across all analyses were corrected to control the false discovery rate via the Benjamini-Hochberg procedure. Adjusted *P* values were evaluated against the significance level of *α* = 0.05. All statistical analyses were carried out using R programming language version 4.4.1 and RStudio for macOS version 2024.09.0 + 375 (Posit Software, Boston, MA, USA).

## RESULTS

### Patient characteristics and exclusions

Twenty-eight patients were originally included in the study between October and December 2021. Twenty-five patients (88% male) finished the study and were included in the analyses, with a median age and dialysis vintage of 66.0 years (48.0, 74.0) and 23.5 months (13.5, 34.5), respectively. Additional baseline patient data are listed in Table 1. Exclusions of patients, bioimpedance measurements, and blood volume estimations are described in the Supplemental Results.

**Table 1:** Baseline patient characteristics.

Variable	*N* = 25
Female sex	3 (12%)
Age, years	66.0 (48.0, 74.0)
Height, m	1.8 (1.7, 1.8)
Cause of KFRT	
Biopsy-proven DKD	1 (4%)
Glomerular	6 (24%)
Not answered	3 (12%)
Other	4 (16%)
Unknown	9 (36%)
Vascular	2 (8%)
Dialysis vintage, months	23.5 (13.5, 34.5)
Albumin, g/l	39.5 (37.7, 41.2)
Total protein, g/l	64.7 (61.6, 67.0)
Hemoglobin, g/dl	10.4 (10.1, 11.2)
Type 1 diabetes	2 (8%)
Type 2 diabetes	9 (36%)
Congestive heart failure	4 (16%)
Arterial hypertension	22 (88%)
Coronary artery disease	14 (56%)
Cerebrovascular disease	5 (20%)
Peripheral arterial disease	5 (20%)
Aortic aneurysm	1 (4%)
History of cancer	6 (24%)

The data are reported as frequency (percentage) or median (quartile 1, quartile 3). Abbreviations: DKD, diabetic kidney disease; KFRT, kidney failure with replacement therapy.

### Time course of analyzed variables

**Figure 1:**
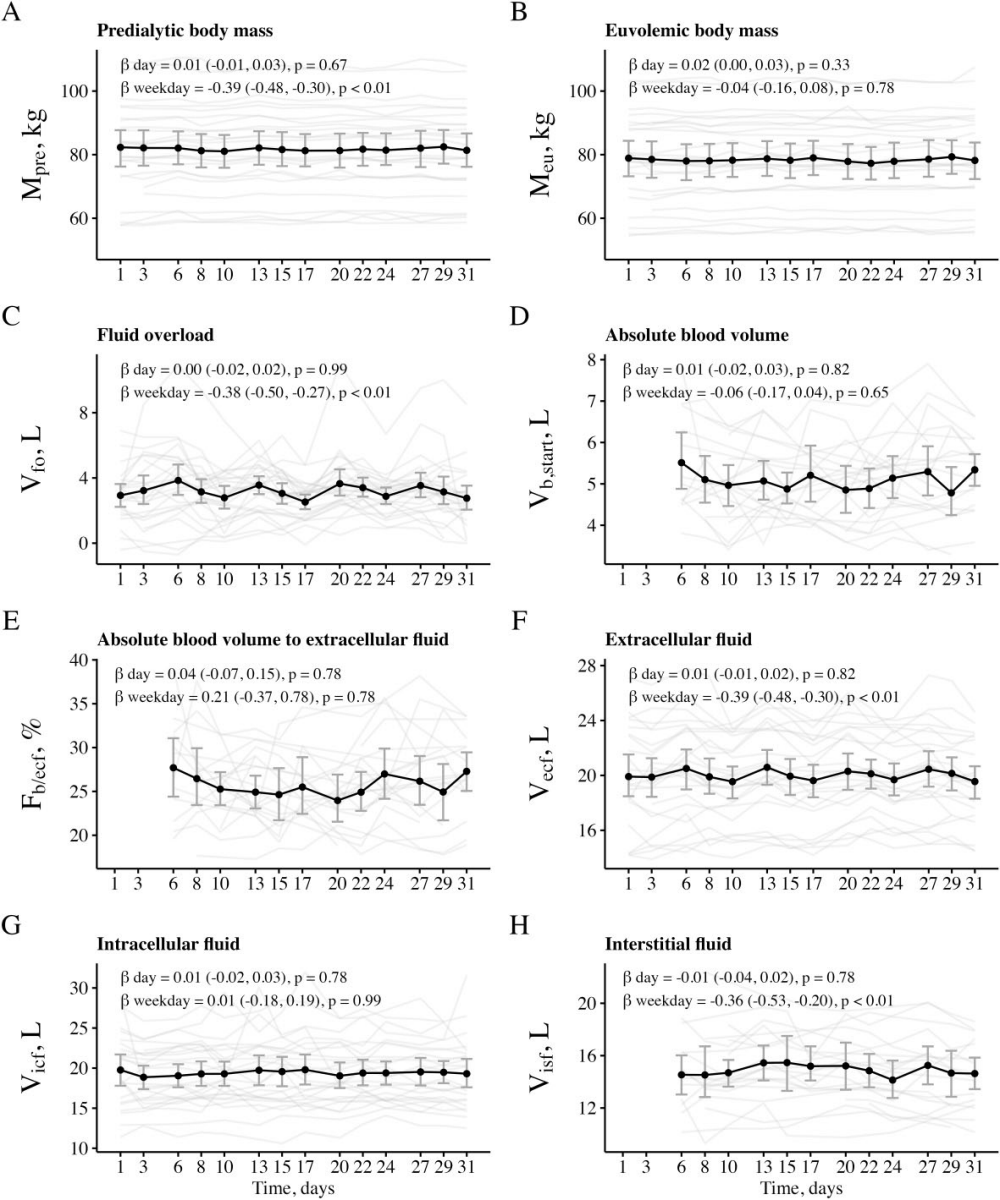
Longitudinal data on pre-dialysis bioimpedance- and blood-volume-derived variables. The data are shown as mean (black dots), bootstrapped 95% confidence interval of the mean (gray error bars) and individual data grouped by patient (light gray lines). *β* coefficients and *P* values from linear mixed models show associations of respective variables with study day (‘*β* day’) and treatment within the week (‘*β* weekday’). Due to the substantial missingness in blood-volume-derived variables (**d, e**, and **h**), the composition of patients varied across study days, which may partly explain some of the observed longitudinal fluctuations in the mean of these variables. Abbreviations: F, fraction; M, body mass; V, volume. Subscripts: b, blood; ecf, extracellular fluid; icf, intracellular fluid; isf, interstitial fluid; eu, euvolemic; fo, fluid overload; pre, pre-dialysis; start, corresponding to treatment start.

**Figure 2:**
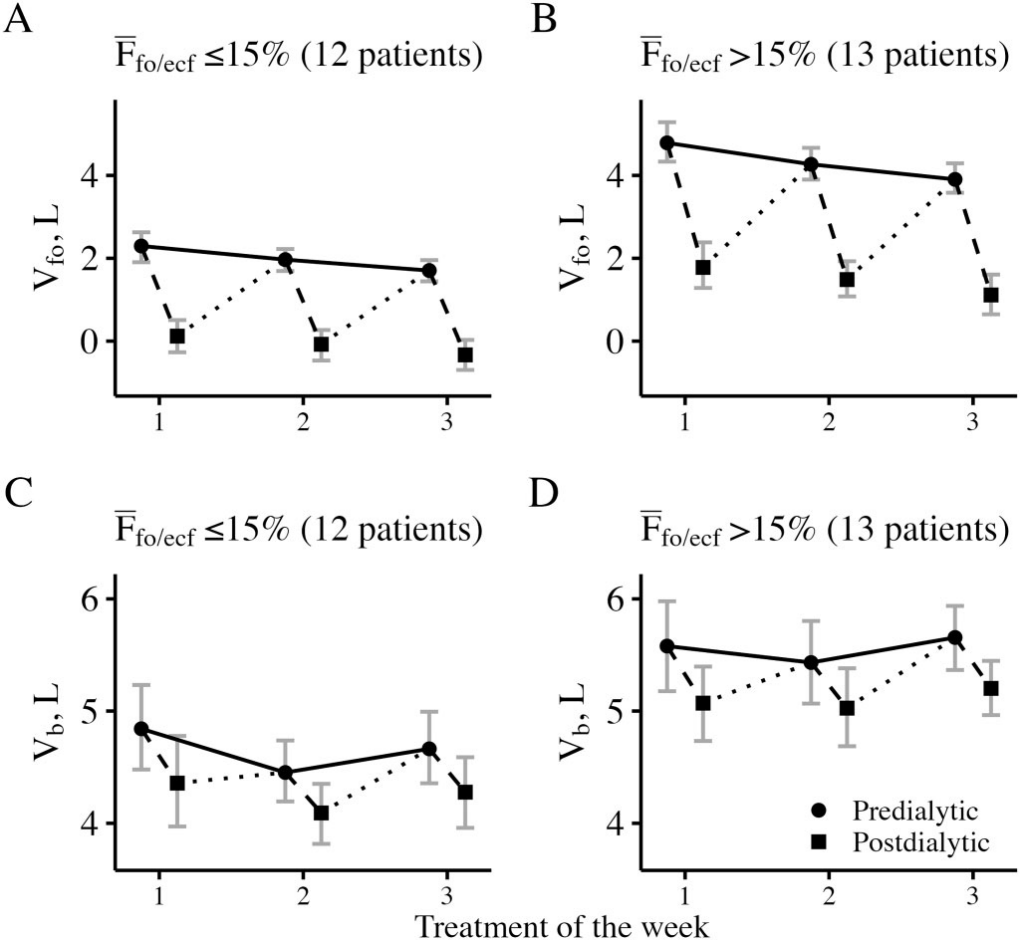
Within-week fluid overload and absolute blood volume. Patients were stratified based on their average predialytic fluid overload relative to the extracellular fluid volume (${{\mathrm{\bar F}}_{{\mathrm{fo}}/{\mathrm{ecf}}}}$ >15% and ${{\mathrm{\bar F}}_{{\mathrm{fo}}/{\mathrm{ecf}}}}$ ≤15%). Dots show mean predialytic values, squares show mean postdialytic values and gray error bars delimit the bootstrapped 95% confidence interval of the mean. Abbreviations: F, fraction; V, volume. Subscripts: b, blood; ecf, extracellular fluid; fo, fluid overload.

The overview of all analyzed variables overall and stratified by treatment within the week are listed in Table 2. Estimates of blood volume were available for 157 treatments with median absolute blood volume of 5.0 l (4.4, 5.7) and specific blood volume of 62.0 ml/kg (52.4, 77.9) at treatment start. The longitudinal patterns in selected variables and the results from linear mixed models are shown in Figs 1 and 2 and Table 3. Pre-dialysis fluid overload showed a decrease during the weekly treatment cycle while pre-dialysis blood volume remained relatively stable on the cohort level. None of the analyzed variables showed an association with study day. Intra-patient coefficients of variation of analyzed variables averaged over the entire study population are listed in Table S1. The mean (±standard deviation) coefficient of variation of euvolemic body mass estimated before dialysis was below 1%, despite relatively high variation in the pre-dialysis fluid overload [coefficient of variation of ∼35% (±30%)]. The ratio between vascular refilling volume and ultrafiltration volume varied to a much higher extent in the initial phase of dialysis treatment compared with the final phase of the treatment [mean coefficient of variation 55.46% (±35.49%) vs. 9.36% (±5.52%)].

**Table 2:** Overview of analyzed variables.

Variable	Unit	Symbol	Observations	Overall, *N* = 350	First treatment, *N* = 100	Second treatment, *N* = 125	Third treatment, *N* = 125
Body mass pre-dialysis	kg	*M*_pre_	345	82.5 (73.0, 91.0)	82.9 (73.2, 91.7)	82.7 (73.0, 91.0)	81.0 (72.5, 91.1)
Body mass post-dialysis	kg	*M*_post_	333	79.3 (70.2, 89.7)	79.4 (70.5, 90.0)	79.4 (70.5, 89.7)	79.0 (69.3, 89.1)
Target body mass	kg	*M*_target_	292	79.0 (70.5, 88.0)	79.0 (70.5, 88.0)	79.0 (71.0, 88.0)	79.0 (70.5, 88.0)
Euvolemic body mass	kg	*M*_eu_	330	77.4 (69.2, 89.4)	77.2 (69.0, 89.5)	77.7 (69.4, 89.1)	77.4 (69.1, 89.5)
Fluid overload pre-dialysis	l	*V*_fo_	330	3.1 (2.1, 4.0)	3.6 (2.3, 4.7)	3.0 (2.1, 4.0)	2.7 (1.6, 3.7)
Relative fluid overload (% extracellular fluid)	%	*F*_fo/ecf_	330	15.1 (11.0, 19.9)	16.8 (12.6, 21.2)	15.1 (12.0, 19.1)	13.8 (9.4, 18.1)
Relative fluid overload >15%			330	165.0 (50.0%)	60.0 (63.8%)	60.0 (50.4%)	45.0 (38.5%)
Extracellular fluid pre-dialysis	l	*V*_ecf_	330	19.9 (17.9, 22.8)	20.4 (18.4, 23.4)	19.8 (17.9, 22.9)	19.7 (17.8, 22.2)
Intracellular fluid pre-dialysis	l	*V*_icf_	330	18.5 (16.2, 22.4)	18.0 (16.2, 22.7)	19.6 (16.2, 22.4)	18.5 (16.1, 21.8)
Total body fluid pre-dialysis	l	*V*_tbf_	330	38.8 (34.1, 44.4)	38.9 (34.6, 45.4)	38.9 (34.1, 44.5)	38.4 (33.6, 43.4)
Interstitial fluid pre-dialysis	l	*V*_isf_	150	14.8 (12.8, 16.7)	15.0 (12.7, 16.6)	14.1 (12.6, 17.1)	14.6 (13.3, 16.5)
Lean tissue mass index pre-dialysis	kg/m^2^		330	12.0 (10.0, 14.8)	12.0 (10.0, 14.8)	12.3 (9.8, 14.9)	11.8 (10.1, 14.4)
Fat tissue mass index pre-dialysis	kg/m^2^		330	13.8 (8.3, 17.8)	14.2 (8.4, 17.9)	13.5 (8.3, 17.4)	13.7 (8.3, 17.9)
Body mass index pre-dialysis	kg/m^2^		330	26.7 (22.6, 29.1)	26.8 (22.8, 29.2)	26.9 (22.6, 29.4)	26.7 (22.3, 28.9)
Treatment duration	min		323	239.1 (211.5, 241.7)	239.7 (211.5, 241.3)	238.2 (211.2, 241.6)	239.7 (212.8, 242.0)
Cumulative ultrafiltration volume	l	*V*_u_	323	2.5 (1.8, 3.1)	2.6 (1.8, 3.5)	2.5 (1.8, 3.0)	2.5 (1.8, 3.0)
Relative blood volume at treatment end	%	*F*_b,end_	320	92.9 (87.7, 97.3)	93.8 (87.7, 97.9)	92.6 (87.5, 97.4)	93.1 (88.3, 96.6)
Refilling volume	l	*V*_r_	157	1.9 (1.2, 2.4)	2.0 (1.4, 2.7)	1.8 (1.0, 2.4)	1.9 (1.1, 2.4)
Blood volume at treatment start	l	*V*_b,start_	157	5.0 (4.4, 5.7)	5.0 (4.4, 6.0)	4.8 (4.2, 5.5)	5.1 (4.5, 5.7)
Blood volume at treatment start per post-dialysis body mass	ml/kg	*V*_b,bm,start_	156	62.0 (52.4, 77.9)	63.3 (53.6, 77.8)	59.5 (51.5, 75.8)	63.9 (52.6, 80.1)
Blood volume at treatment start per lean tissue mass	ml/kg	*V*_b,lt,start_	150	135.2 (116.5, 160.9)	135.6 (119.8, 163.1)	134.1 (116.5, 154.4)	133.5 (112.0, 160.0)
Ultrafiltration rate during the first 30 min	ml/min	*Q*_u,start_	322	10.0 (9.0, 14.0)	10.0 (7.9, 16.0)	10.0 (9.9, 14.0)	10.0 (9.0, 13.1)
Refilling rate during the first 30 min	ml/min	*Q*_r,start_	157	7.3 (4.8, 10.0)	7.1 (5.2, 9.4)	7.2 (3.5, 10.4)	7.3 (5.2, 10.0)
Refilling fraction based on rates during the first 30 min	%	*F*_r/u,q,start_	156	74.2 (54.2, 96.8)	70.8 (52.5, 93.0)	75.4 (59.1, 100.0)	75.9 (48.2, 99.6)
Refilling fraction based on volumes during the first 30 min	%	*F*_r/u,v,start_	153	62.2 (39.2, 89.7)	62.1 (30.9, 84.7)	59.2 (25.2, 92.9)	71.2 (43.8, 89.8)
Blood volume to extracellular fluid at treatment start	%	*F*_b/ecf_	150	25.5 (22.3, 29.1)	24.2 (22.3, 29.0)	25.8 (21.1, 28.8)	25.7 (23.2, 30.1)
Blood volume at treatment end	l	*V*_b,end_	157	4.7 (4.0, 5.2)	4.7 (3.9, 5.2)	4.5 (3.9, 5.0)	4.8 (4.1, 5.4)
Blood volume at treatment end per post-dialysis body mass	ml/kg	*V*_b,bm,end_	156	55.6 (48.1, 70.9)	56.0 (48.6, 69.1)	53.6 (45.9, 69.9)	56.1 (49.5, 77.4)
Blood volume at treatment end per lean tissue mass	ml/kg	*V*_b,lt,end_	150	124.6 (104.3, 149.7)	123.9 (107.0, 144.0)	125.2 (102.7, 149.7)	124.2 (105.5, 150.5)
Ultrafiltration rate during the last 30 min	ml/min	*Q*_u,end_	322	10.0 (8.5, 13.8)	10.0 (8.0, 16.0)	10.0 (9.5, 13.1)	10.0 (8.5, 12.0)
Refilling rate during the last 30 min	ml/min	*Q*_r,end_	157	9.6 (6.1, 11.9)	9.9 (6.7, 12.6)	9.8 (5.4, 11.1)	9.2 (6.1, 11.8)
Refilling fraction based on rates during the last 30 min	%	*F*_r/u,q,end_	155	87.3 (77.8, 97.2)	87.1 (78.9, 94.7)	89.3 (77.8, 100.0)	84.2 (76.9, 96.7)
Refilling fraction based on volumes during the last 30 min	%	*F*_r/u,v,start_	156	71.0 (65.0, 77.3)	70.8 (66.8, 76.0)	70.7 (62.2, 77.9)	72.3 (64.4, 78.5)
Systolic blood pressure pre-dialysis	mmHg		286	142 (127, 157)	141 (127, 157)	142 (129, 156)	143 (124, 158)
Systolic blood pressure post-dialysis	mmHg		251	139 (119, 157)	138 (125, 160)	143 (117, 159)	139 (119, 154)
Diastolic blood pressure pre-dialysis	mmHg		286	72 (61, 81)	71 (63, 79)	71 (62, 81)	73 (60, 82)
Diastolic blood pressure post-dialysis	mmHg		251	72 (61, 82)	73 (61, 84)	72 (64, 82)	72 (56, 82)
Treatments with intradialytic hypotension			292	27 (9.2%)	8 (8.2%)	7 (7.2%)	12 (12.4%)

The data are reported as median (quartile 1, quartile 3) or frequency (percentage) and are provided both for all dialysis treatments from the entire study and stratified by treatment within the week. Not all data were available from all dialysis treatments (especially the data on blood volume and vascular refilling), and hence the number of observations (*N*) are lower from the number of all studied treatments.

**Table 3:** Parameter estimates of linear mixed models for study day and treatment within the week.

	IV: Study day			IV: Treatment within the week	
DV	*β* (95% CI)	*P*	SD slope	*β* (95% CI)	*P*
*M*_pre_, kg	0.01 (−0.01, 0.03)	.67	0.04	−0.39 (−0.48, −0.30)	<.01
*M*_eu_, kg	0.02 (0.00, 0.03)	.33	0.03	−0.04 (−0.16, 0.08)	.78
*V*_fo_, l	0.00 (−0.02, 0.02)	.99	0.04	−0.38 (−0.50, −0.27)	<.01
*F*_fo/ecf_, %	0.01 (−0.07, 0.08)	.96	0.15	−1.54 (−2.02, −1.06)	<.01
*V*_tbf_, l	0.01 (−0.03, 0.05)	.78	0.08	−0.38 (−0.61, −0.16)	<.01
*V*_ecf_, l	0.01 (−0.01, 0.02)	.82	0.05	−0.39 (−0.48, −0.30)	<.01
*V*_icf_, l	0.01 (−0.02, 0.03)	.78	0.05	0.01 (−0.18, 0.19)	.99
*V*_isf_, l	−0.01 (−0.04, 0.02)	.78	0.06	−0.36 (−0.53, −0.20)	<.01
*F*_b,end_, %	0.03 (−0.03, 0.09)	.67	0.12	0.11 (−0.36, 0.57)	.83
*V*_b,start_, l	0.01 (−0.02, 0.03)	.82	0.04	−0.06 (−0.17, 0.04)	.65
*V*_b,end_, l	0.01 (−0.02, 0.03)	.82	0.04	−0.05 (−0.15, 0.06)	.74
*V*_b,bm,start_, ml/kg	0.09 (−0.19, 0.37)	.78	0.56	−0.42 (−1.77, 0.92)	.78
*V*_b,bm,end_, ml/kg	0.10 (−0.18, 0.37)	.78	0.54	−0.27 (−1.62, 1.09)	.86
*V*_b,lt,start_, ml/kg	0.00 (−0.73, 0.74)	.99	1.39	−2.88 (−6.96, 1.21)	.63
*V*_b,lt,end_, ml/kg	0.04 (−0.69, 0.76)	.99	1.39	−2.77 (−6.76, 1.21)	.63
*F*_b/ecf,start_, %	0.04 (−0.07, 0.15)	.78	0.22	0.21 (−0.37, 0.78)	.78
*V*_u_, l	0.00 (−0.01, 0.01)	.94	NA	−0.12 (−0.19, −0.05)	<.01
*V*_r_, l	0.00 (−0.01, 0.00)	.74	NA	−0.12 (−0.21, −0.04)	.05
*Q*_u,start_, ml/min	−0.02 (−0.09, 0.06)	.85	0.16	−0.27 (−0.75, 0.21)	.67
*Q*_u,end_, ml/min	0.00 (−0.06, 0.06)	.99	0.11	−0.67 (−1.17, −0.17)	.06
*Q*_r,start_, ml/min	0.00 (−0.06, 0.06)	.99	NA	−0.04 (−0.62, 0.54)	.99
*Q*_r,end_, ml/min	0.01 (−0.05, 0.07)	.96	NA	−0.51 (−1.07, 0.06)	.36
*F*_r/u,q,start_, %	−0.20 (−0.63, 0.22)	.74	NA	0.30 (−3.79, 4.39)	.99
*F*_r/u,q,end_, %	0.13 (−0.19, 0.45)	.77	NA	−2.94 (−6.08, 0.20)	.33
*F*_r/u,v,start_, %	−0.69 (−1.62, 0.23)	.63	1.53	0.90 (−5.43, 7.22)	.92
*F*_r/u,v,end_, %	−0.06 (−0.20, 0.09)	.77	NA	−0.77 (−2.16, 0.62)	.67

Dependent variables were modeled by linear mixed models with study day and treatment within the week as fixed effects (both in the same model), with random intercepts and random slopes for study day per patient. Age and sex were added to the model as confounders. “*β* (95% CI)” denotes the effect estimates of the fixed effects, *P* denotes *P* values of the fixed effects and “SD slope” is the standard deviation of the random slopes of study day in all patients. If SD slope is NA, the model was fit without random slopes. Abbreviations: CI, confidence interval; DV, dependent variable; F, fraction; IV, independent variable; M, mass; Q, rate; SD, standard deviation; V, volume. Subscripts: b, blood; bm, normalized to post-dialysis body mass; ecf, extracellular fluid; end, corresponding to treatment end; eu, euvolemic; icf, intracellular fluid; isf, interstitial fluid; fo, fluid overload; lt, normalized to lean tissue mass; post, post-dialysis; pre, pre-dialysis; r, refilling; start, corresponding to treatment start; tbf, total body fluid; u, ultrafiltration.

### Repeated measures correlations

**Figure 3:**
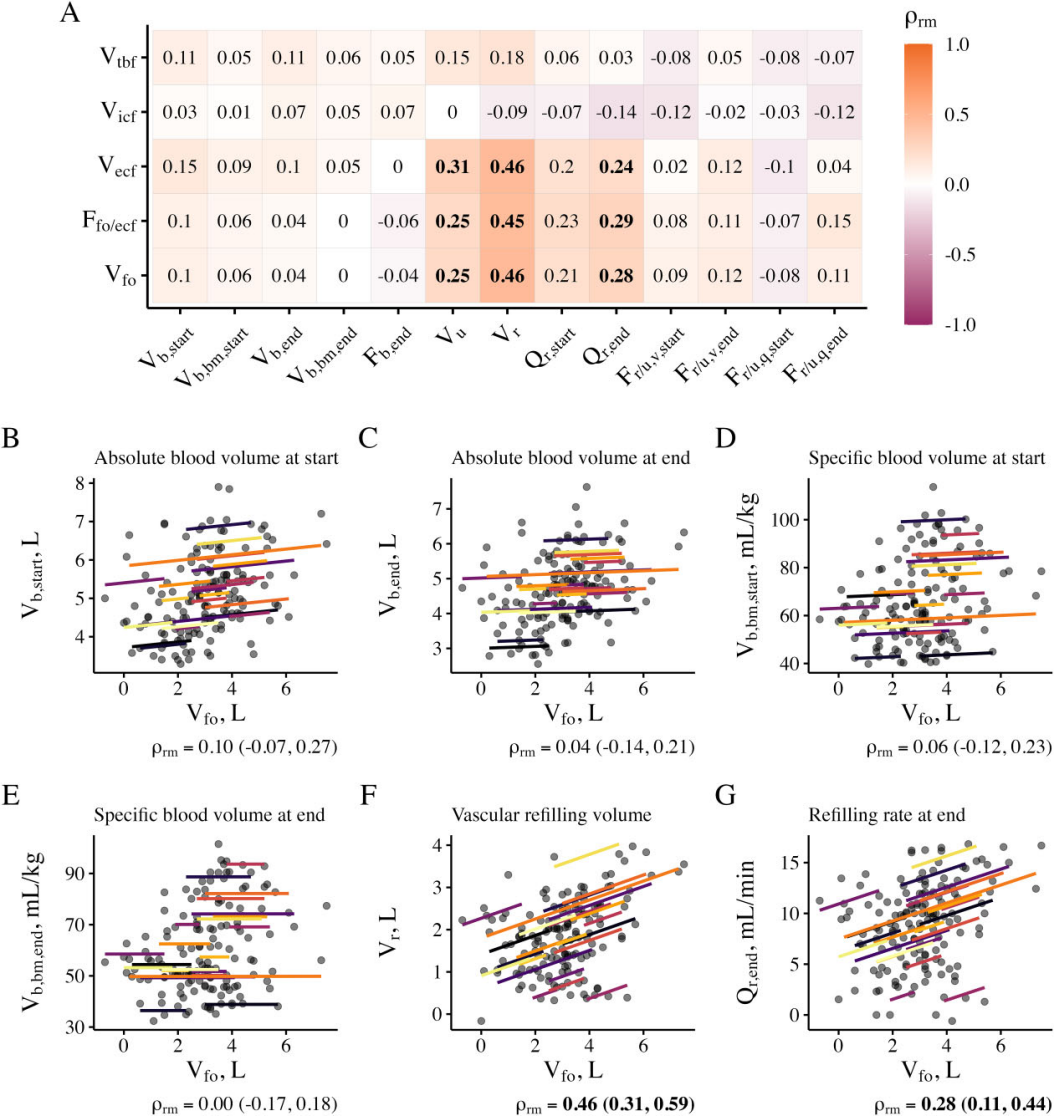
Coefficients of repeated measures correlations between the analyzed variables. (**A**) Coefficients of repeated measures correlation between the analyzed variables (${\rho _{rm}}$) in matrix form. The color indicates the strength of correlation. (**B**–**G**) Correlations between the volume of pre-dialysis fluid overload (*V*_fo_) and selected variables. Each line and color represents one patient. ${\rho _{rm}}$ are reported with 95% confidence intervals. Coefficients printed in bold represent statistically significant correlations. Abbreviations: F, fraction; Q, rate; V, volume. Subscripts: b, blood; bm, postdialytic body mass; ecf, extracellular fluid; end, corresponding to treatment end; icf, intracellular fluid; fo, fluid overload; q, fraction calculated from rates (of refilling and ultrafiltration); r, refilling; start, corresponding to treatment start; tbf, total body fluid; u, ultrafiltration; v, fraction calculated from volumes (of refilling and ultrafiltration).

Intra-patient repeated measures correlations between ultrafiltration volumes and rates, bioimpedance-derived fluid volumes, blood volume, and vascular refilling variables are shown in Fig. 3 and Table S2. Neither absolute nor relative fluid overload were significantly correlated with absolute or specific blood volume at the start or end of the treatment on the intra-patient level. Pre-dialysis fluid overload exhibited a moderate correlation with the total refilling volume [${\rho _{rm}}$= 0.46 (95% CI 0.31, 0.59), *P* < .01], which was stronger than with ultrafiltration volume [${\rho _{rm}}$= 0.25 (95% CI 0.14, 0.36), *P* < .01]. Intracellular fluid volume showed no statistically significant correlation with any blood-volume-derived variable.

### Intradialytic blood volume and vascular refilling

**Figure 4:**
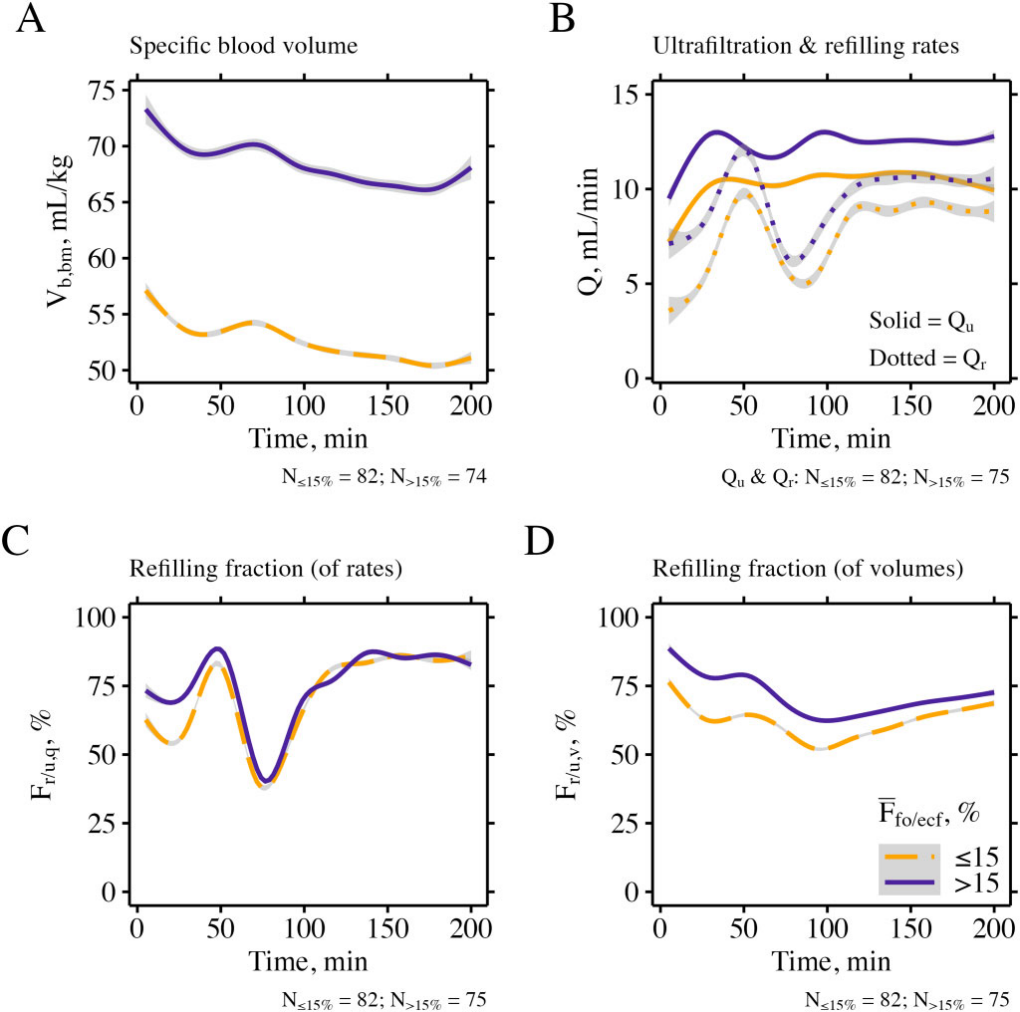
Intradialytic changes in specific blood volume, ultrafiltration and vascular refilling. Patients were stratified based on their average predialytic fluid overload relative to the extracellular fluid volume [${{\mathrm{\bar F}}_{{\mathrm{fo}}/{\mathrm{ecf}}}}$ ≤15% (orange dashed line, 12 patients) and ${{\mathrm{\bar F}}_{{\mathrm{fo}}/{\mathrm{ecf}}}}$ >15% (violet solid line, 13 patients)]. Data from all treatments of the respective patient group were fitted by a non-linear smooth function (method “gam”). Shaded areas delimit the 95% confidence interval. Notice that the disturbance starting around 60 min is caused by the infusion of dialysate to measure absolute blood volume. Time was cut off at 200 min to avoid distortion due to fewer data at later time points. Data on blood volume from the first 5 min of treatment and within 1 min before to 5 min after the dialysate bolus infusion, refilling fractions >200% or <−100% as well as refilling rates <−50 ml/min were removed from analysis. Abbreviations: F, fraction; Q, flow rate; V, volume. Subscripts: b, blood; bm, normalized to post-dialysis body mass; ecf, extracellular fluid; fo, fluid overload; q, refilling fraction calculated from rates (of refilling and ultrafiltration); r, refilling; u, ultrafiltration; v, refilling fraction calculated from volumes (of refilling and ultrafiltration).

The intradialytic time course of specific blood volume, ultrafiltration rate, and vascular refilling measures, stratified by average pre-dialysis fluid overload above or below 15% of extracellular fluid are shown in Fig. 4. Patients with average pre-dialysis relative fluid overload >15% exhibited higher specific blood volume, higher ultrafiltration rates, and higher refilling rates, but only marginally higher refilling fractions compared to patients in whom average pre-dialysis relative fluid overload was ≤15%. The increase in specific blood volume and concomitant drops in refilling rate and refilling fractions starting ∼60 min coincided with the infusion of the dialysate bolus for the estimation of blood volume. These variables leveled off after ∼120 min of treatment when investigated per treatment (Figs S1–S5). Patient and treatment characteristics at the time of recorded intradialytic hypotension are listed in Table S3, and intradialytic patterns of blood volume, ultrafiltration and vascular refilling stratified by the presence or absence of intradialytic hypotension are shown in Fig. S6. Specific blood volume at treatment start was <65 ml/kg in 72% of treatments with hypotensive episodes and available blood volume data compared with 58% of treatments without intradialytic hypotension.

## DISCUSSION

The main result of this study examining the within-week and week-to-week variability of fluid status in maintenance hemodialysis patients is the relative stability of pre-dialysis intracellular fluid volume and euvolemic body mass and the lack of a clear within-week pattern in pre-dialysis blood volume. By contrast, fluid overload and extracellular fluid volume exhibited a regular pattern of decline during weekly treatment cycles, with a return to baseline after each cycle as described previously [24–27]. This is, to our knowledge, the first study to simultaneously capture bioimpedance-derived fluid overload and blood volume data from multiple consecutive dialysis treatments over a prolonged period of four weeks.

Because of the well-established one-to-three ratio between blood volume and extracellular fluid volume [28], one might have expected a similar sawtooth-like pattern for blood volume. Surprisingly, such a pattern was not found in our study. Pre-dialysis fluid overload did not correlate with blood volume, suggesting that fluid overload did not accumulate proportionally in the interstitial space, which could be serving as a buffer for excess fluid volume. Kron *et al.* previously found a correlation between pre-dialysis fluid overload and specific blood volume at treatment start in stable patients (*r* = 0.45, *P* < .01) but not in patients prone to intradialytic morbid events (*r* = 0.378, not significant) [29]. In another cohort, they found a correlation between pre-dialysis fluid overload and specific blood volume (*r* = 0.42, *P* < .05) [15]. However, their studies did not investigate intra-patient correlations but only cohort-wide correlations. Note that both blood volume [30, 31] and whole-body bioimpedance-derived variables [7, 8] scale with body mass and body height. Therefore, a positive correlation, as found by Kron *et al*., is to be expected when studying a heterogeneous group of different body sizes. Koomans *et al.* [32] previously described patterns of plasma refilling, where plasma volume reached a nadir at the end of ultrafiltration but reverted, on average, close to initial conditions after 24 hours post-treatment. However, they observed that the level of plasma refilling 24 hours post-treatment depended on the pre-dialysis fluid overload, with a return of plasma volume close to the pre-dialysis level only in highly fluid overloaded patients (possibly even with an overshoot of plasma volume) and an incomplete plasma refilling in patients with low or moderate pre-dialysis fluid overload. A similar conclusion with regard to patients with low and moderate fluid overload could be drawn from the study by Kron *et al.* [15], although they studied the level of plasma refilling only at the end of treatment. While our data may suggest a setpoint theory for blood volume at the cohort level, large intra-patient variability complicates drawing the same conclusion at the individual level. Note that the coefficient of variation for repeated measurements was ∼5% for total body fluid volume, as well as intra- and extracellular fluid, but ∼10% for absolute and specific blood volumes. We stress that our hypothesis on the non-linear relationship between pre-dialysis blood volume and fluid overload must be confirmed by increasing the accuracy of pre-dialysis blood volume estimations and ideally by studying interventions where larger changes in blood volume would be provoked.

In our study, cumulative intradialytic refilling volume showed a moderate correlation with pre-dialysis fluid overload. Alvarez-Nadal *et al.* [16] reported a stronger correlation between intradialytic refilling volume and interdialytic gain in body mass (*r*^2 ^= 0.617), which they used as a surrogate for fluid overload. Contrary to these results, Kron *et al.* did not find any correlation between intradialytic refilling volume and fluid overload (*r* = 0.05, *P* = .79) [15]. The intradialytic refilling rates in our study were higher in patients with average pre-dialysis relative fluid overload >15% compared to those ≤15%, which is plausible since ultrafiltration volume is typically higher in patients with increased apparent fluid excess. Mitsides *et al.* found a strong association between pre-dialysis fluid overload and intradialytic refilling rate in a linear model that was also adjusted for ultrafiltration rate [17]. We were not able to obtain similar results with our refilling data from the first 30 min of treatments. The variability of refilling measures during the initial 30 min of treatment was high, as reported in other studies [17, 33]. The refilling rate at the end of treatment, however, was relatively stable, and the average refilling rate during the last 30 min of treatment was correlated with pre-dialysis fluid overload. The refilling fraction (i.e. the percentage of ultrafiltered volume compensated by the refilling volume) was only slightly higher in the fluid overloaded group but was not associated with pre-dialysis fluid overload neither within the first nor last 30 min of treatment.

This study is limited by the cohort size, the predominance of males and the exploratory nature of the analysis. However, the longitudinal design providing repeated measures in the same patients under similar conditions accounting for the variability of unevenly spaced treatments and the use of combined bioimpedance measurements and blood volume estimations are notable strengths. Hypotheses generated in this study must be evaluated in separate, larger-scale trials. A *post hoc* power analysis revealed that our sample sizes permitted detecting a 250-ml change with each subsequent within-week treatment in pre-dialysis fluid overload or blood volume with a power of 99.2% and 99.5%. One-third of the blood volume estimates had to be excluded, mainly because the unusual shape of the relative blood volume curves or their lack of stability did not allow for a reliable application of the method [14], potentially limiting generalizability of our results. Estimated vascular refilling may have been slightly affected by possible osmotic water shifts between plasma and red blood cells in treatments performed with DBB-EXA machines, which use optical measurements of infrared light reflected from red blood cells to track relative blood volume during dialysis [34]. Bioimpedance measurements may have been somewhat affected by potential variation in pre-dialysis tissue sodium concentration, which affects tissue conductance and thereby fluid volume estimation [35], although while an intra-dialysis decline in tissue sodium was previously observed [36], changes in pre-dialysis tissue sodium throughout the weekly treatment cycle remain unknown. We intentionally abstained from using anthropometric equations for blood volume since these operate under the assumption that changes in body mass (and fluid status) have an effect on blood volume, which we wanted to investigate in the first place.

In conclusion, our study found that the estimated pre-dialysis absolute blood volume did not exhibit the expected sawtooth-like pattern throughout multiple treatment cycles (as pre-dialysis fluid overload does) and showed unsystematic variability on an intra-patient level, thus challenging previous assumptions. The lack of correlation between pre-dialysis absolute blood volume and fluid overload suggests that fluid expansion in maintenance hemodialysis patients may occur primarily in the interstitial space. Additionally, we presented continuous intradialytic data on vascular refilling in conjunction with pre-dialysis fluid status across multiple dialysis treatments, thus enhancing existing knowledge on the intradialytic dynamics of vascular refilling.

## Supplementary Material

sfaf199_Supplemental_File

## Data Availability

Consent for public disclosure of individual patient data was not obtained at the time of data collection. Data can therefore not be shared on a public repository.
