# Supplementary material for: Longitudinal patterns of fluid overload, blood volume and vascular refilling: a prospective study in patients on maintenance hemodialysis
Source: Clin Kidney J. 2025 Jun 27;18(8):sfaf199. doi: 10.1093/ckj/sfaf199 (PMC12314271; doi:10.1093/ckj/sfaf199)
Supplement: sfaf199_Supplemental_File [file sfaf199_supplemental_file.docx]

**Supplemental Material to:**

**Longitudinal Patterns of Fluid Overload, Blood Volume and Vascular Refilling: a Prospective Study in Patients on Maintenance Hemodialysis**

Sebastian Mussnig, Simon Krenn, Max Waller, Michael Schmiedecker, Amelie Kurnikowski, Janosch Niknam, Luis Naar, Christopher C. Mayer, David Keane, Daniel Schneditz, Manfred Hecking, Leszek Pstras

**Table of Contents:**

p.2 Supplemental Methods 1: Estimation of blood volume variables

p.4 Supplemental Methods 2: Post-hoc power analysis

p.5 Supplemental Results: Exclusions

p.6 Table S1: Coefficients of variation

p.8 Table S2: Repeated measures correlations

p.10 Table S3: Patient and treatment characteristics during intradialytic hypotension

p.11 Figure S1: Intradialytic changes of specific blood volume grouped by patient and treatment

p.12 Figure S2: Intradialytic changes of ultrafiltration rates grouped by patient and treatment

p.13 Figure S3: Intradialytic changes of vascular refilling rates grouped by patient and treatment

p.14 Figure S4: Intradialytic changes of the ratio between vascular refilling rate and ultrafiltration rate grouped by patient and treatment

p.15 Figure S5: Intradialytic changes of the ratio between vascular refilling volume and ultrafiltration volume grouped by patient and treatment

p.16 Figure S6: Intradialytic changes of specific blood volume, ultrafiltration and vascular refilling

Correspondence to:

Manfred Hecking (manfred.hecking@meduniwien.ac.at)

Medical University of Vienna
Center for Public Health
Department of Epidemiology
Kinderspitalgasse 15, 1^st^ floor
1090 Vienna, Austria

**Supplemental Methods 1: Estimation of blood volume and related variables**

Absolute blood volume at 100% relative blood volume (which roughly corresponds to treatment start [V_b,start_, in mL]) was estimated using an on-line infusion of a dialysate bolus of known volume (V_bolus_, in mL) around 60 min into the dialysis treatment and recorded values of relative blood volume right before and right after bolus infusion (F_b,pre-bolus_ and F_b,post-bolus_, in %) to estimate the bolus-induced increase in blood volume, according to the algorithm described in Pstras et al. (Biomedical Signal Processing and Control. 2024) with some modifications mentioned in the main manuscript.

|  | $V_{b,start}=\frac{V_{\mathrm{bolus}}}{{F_{b,post-bolus}-F}_{b,pre-bolus}}\times100$ | 1 |
| --- | --- | --- |

The above equation is based on a single-compartment blood volume assumption. Absolute blood volume at time t during the treatment (V_b,t_, in mL) can then be computed from V_b,start_ and relative blood volume at time t (F_b,t_, in %) using the following equation.

|  | $V_{b,t}=V_{b,start}\times F_{b,t}\times\frac{1}{100}$ | 2 |
| --- | --- | --- |

V_b,t_ may then be normalized to post-dialysis body mass (M_post_, in kg), resulting in specific blood volume at time t (V_b,bm,t_, in mL/kg). In the case of missing data, post-dialysis body mass was approximated as pre-dialysis body mass minus the total ultrafiltration volume (assuming that 1 L of ultrafiltration volume equals 1 kg).

|  | $V_{b,bm,t}=\frac{V_{b,t}}{M_{\mathrm{post}}}$ | 3 |
| --- | --- | --- |

In the single-compartment blood volume assumption, the cumulative volume of vascular refilling from the extravascular space at time t (V_r,t_, in mL) is given as the cumulative ultrafiltration volume at time t (V_u,t_, in mL) and the difference between V_b,t_ and V_b,start_ corrected for the dialysate bolus infusion by subtracting the cumulative volume of infused dialysate at time t (V_bolus,t_, in mL).

|  | $V_{r,t}=V_{u,t}+\left( V_{b,t}-V_{b,start} \right)-V_{bolus,t}$ | 4 |
| --- | --- | --- |

The ultrafiltration rate at time t (Q_u,t_, in mL/min) is the change in V_u_ from t minus 1 min to t.

|  | $Q_{u,t}={V_{u,t}-V}_{u,(t-1min)}$ | 5 |
| --- | --- | --- |

The refilling rate at time t (Q_r,t_, in mL/min) is the change in V_r_ from t minus 1 min to t.

|  | $Q_{r,t}={V_{r,t}-V}_{r,(t-1min)}$ | 6 |
| --- | --- | --- |

Finally, the refilling fraction of the ultrafiltration rate is calculated as the time-dependent ratio between Q_r,t_ and Q_u,t_ (F_r/u,q,t_, in %) and the refilling fraction of ultrafiltration volume as the time-dependent ratio between V_r,t_ and V_u,t_ (F_r/u,v,t_, in %).

|  | $F_{r/u,t}=\frac{Q_{r,t}}{Q_{u,t}}\times100$ | 7 |
| --- | --- | --- |
|  | $F_{r/u,t}=\frac{V_{r,t}}{V_{u,t}}\times100$ | 8 |

**Supplemental Methods 2: Post-hoc power analysis**

We set the clinically relevant effects of treatment within the week on pre-dialysis fluid overload and blood volume at treatment start to 250 mL. We analyzed the power of our mixed models with the respective clinically relevant fixed effect of weekday using the *PowerSim* function from the *simr* library, setting the number of simulations to 1000. In our sample, an effect of 250 mL per weekday on pre-dialysis fluid overload could be detected with a power of 99.20% (98.43, 99.65). The power of detecting a 250 mL effect of weekday on blood volume at treatment start was 99.50% (98.84, 99.84). The analysis code is provided below.

**
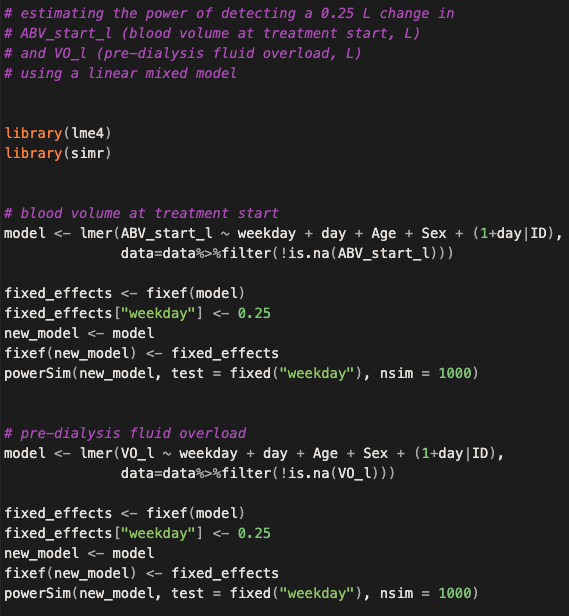
**

**Supplemental Results: Exclusions**

Twenty-eight patients were originally included in the study. One patient initially experienced frequent intradialytic morbid events and therefore discontinued study participation, was later included again, but died shortly after due to causes unrelated to the study. One patient suffered from shunt thrombosis early during the study, rendering subsequent participation impossible. One patient revoked participation during the second dialysis treatment. Twenty-five patients finished the study and were included in the analyses.

Bioimpedance spectroscopy measurements were scheduled for 350 treatments, and 330 measurements were available for analysis (20 exclusions: 7 quality index <85%; 6 implausible input body mass; 7 the device did not give an estimate of fluid overload). Absolute blood volume estimations were scheduled in 300 treatments, and 157 estimates were available for analysis (143 exclusions: 18 no relative blood volume data; 16 no bolus infusion; 2 bolus infusion not recorded; 96 issue with unusual shape or lack of stability of relative blood volume curves, questioning whether the relative increase in blood volume observed after the bolus infusion was caused solely by the bolus, e.g. relative blood volume staying at an elevated level after the bolus infusion instead of decreasing, an inconsistent relative blood volume profile before and after the bolus infusion, or dynamic changes of blood volume right before the bolus infusion; 11 implausible blood volume estimates, considered as values below 3 L or above 8 L, or values implausible for the given patient given the patient’s size and blood volume estimates from other treatments).

**Supplemental Tables**

Table S1: Coefficients of variation of analyzed variables

| **Variable** | **Mean CV (%)** | **SD CV (%)** |
| --- | --- | --- |
| M_pre_ | 0.99 | 0.46 |
| M_post_ | 0.91 | 0.61 |
| M_target_ | 0.27 | 0.66 |
| M_eu_ | 1.09 | 0.54 |
| V_fo_ | 34.86 | 28.83 |
| F_fo/ecf_ | 32.14 | 30.02 |
| V_tbf_ | 4.03 | 1.81 |
| V_ecf_ | 4.03 | 1.37 |
| V_icf_ | 5.70 | 3.91 |
| V_isf_ | 6.25 | 2.36 |
| V_u_ | 19.90 | 9.14 |
| V_r_ | 24.64 | 15.56 |
| F_b,end_ | 3.68 | 1.38 |
| V_b,start_ | 10.66 | 5.79 |
| V_b,bm,start_ | 10.63 | 5.66 |
| V_b,lt,start_ | 13.02 | 6.70 |
| Q_u,start_ | 38.13 | 20.83 |
| Q_r,start_ | 36.70 | 17.30 |
| F_r/u,q,start_ | 26.89 | 17.43 |
| F_r/u,v,start_ | 55.46 | 35.49 |
| F_b/ecf,start_ | 11.11 | 5.32 |
| V_b,end_ | 11.61 | 6.36 |
| V_b,bm,end_ | 11.53 | 6.29 |
| V_b,lt,end_ | 13.89 | 6.80 |
| Q_u,end_ | 39.85 | 24.27 |
| Q_r,end_ | 34.87 | 21.68 |
| F_r/u,q,end_ | 16.14 | 12.17 |
| F_r/u,v,end_ | 9.36 | 5.52 |

Legend to Table S1: For each variable, coefficients of variation were calculated per patient and subsequently averaged over the entire study population. Abbreviations: CV, coefficient of variation; F, fraction; M, body mass; SD, standard deviation; Q, rate; V, volume. Subscripts: b, relative blood volume at treatment end; bm, normalized to post-dialysis body mass; ecf, extracellular fluid; end, corresponding to treatment end; eu, euvolemic; icf, intracellular fluid; isf, interstitial fluid; lt, normalized to lean tissue mass; fo, fluid overload; post, post-dialysis; pre, pre-dialysis; q, refilling fraction calculated from rates (of refilling and ultrafiltration); r, refilling; start, corresponding to treatment start; tbf, total body fluid; u, ultrafiltration; v, refilling fraction calculated from volumes (of refilling and ultrafiltration).

Table S2: Repeated measures correlations between analyzed variables

| **Variable 1** | **Variable 2** | **df** | $\boldsymbol{\rho}_{\boldsymbol{rm}}$ | **95% CI** | ***P*** |
| --- | --- | --- | --- | --- | --- |
| V_fo_ | V_r_ | 124 | 0.46 | ( 0.31, 0.59) | <0.01 |
| V_ecf_ | V_r_ | 124 | 0.46 | ( 0.31, 0.59) | <0.01 |
| F_fo/ecf_ | V_r_ | 124 | 0.45 | ( 0.30, 0.58) | <0.01 |
| V_ecf_ | V_u_ | 281 | 0.31 | ( 0.20, 0.41) | <0.01 |
| F_fo/ecf_ | V_u_ | 281 | 0.25 | ( 0.14, 0.36) | <0.01 |
| V_fo_ | V_u_ | 281 | 0.25 | ( 0.14, 0.36) | <0.01 |
| F_fo/ecf_ | Q_r,end_ | 124 | 0.29 | ( 0.13, 0.45) | <0.01 |
| V_fo_ | Q_r,end_ | 124 | 0.28 | ( 0.11, 0.44) | 0.01 |
| V_ecf_ | Q_r,end_ | 124 | 0.24 | ( 0.07, 0.40) | 0.05 |
| F_fo/ecf_ | Q_r,start_ | 124 | 0.23 | ( 0.06, 0.39) | 0.06 |
| V_tbf_ | V_u_ | 281 | 0.15 | ( 0.03, 0.26) | 0.08 |
| V_fo_ | Q_r,start_ | 124 | 0.21 | ( 0.04, 0.38) | 0.09 |
| V_ecf_ | Q_r,start_ | 124 | 0.20 | ( 0.03, 0.36) | 0.13 |
| V_tbf_ | V_r_ | 124 | 0.18 | ( 0.01, 0.35) | 0.20 |
| V_ecf_ | V_b,start_ | 124 | 0.15 | (-0.02, 0.32) | 0.37 |
| F_fo/ecf_ | F_r/u,q,end_ | 122 | 0.15 | (-0.02, 0.32) | 0.38 |
| V_icf_ | Q_r,end_ | 124 | -0.14 | (-0.30, 0.04) | 0.52 |
| V_fo_ | F_r/u,v,end_ | 123 | 0.12 | (-0.06, 0.29) | 0.63 |
| V_ecf_ | F_r/u,v,end_ | 123 | 0.12 | (-0.06, 0.29) | 0.63 |
| V_icf_ | F_r/u,v,start_ | 120 | -0.12 | (-0.29, 0.06) | 0.63 |
| V_icf_ | F_r/u,q,end_ | 122 | -0.12 | (-0.29, 0.06) | 0.63 |
| F_fo/ecf_ | F_r/u,v,end_ | 123 | 0.11 | (-0.06, 0.28) | 0.65 |
| V_fo_ | V_b,start_ | 124 | 0.10 | (-0.07, 0.27) | 0.65 |
| V_fo_ | F_r/u,q,end_ | 122 | 0.11 | (-0.07, 0.28) | 0.65 |
| V_ecf_ | V_b,end_ | 124 | 0.10 | (-0.07, 0.27) | 0.65 |
| V_ecf_ | F_r/u,q,start_ | 123 | -0.10 | (-0.27, 0.07) | 0.65 |
| V_icf_ | F_b,end_ | 278 | 0.07 | (-0.05, 0.19) | 0.65 |
| V_tbf_ | V_b,start_ | 124 | 0.11 | (-0.07, 0.28) | 0.65 |
| V_tbf_ | V_b,end_ | 124 | 0.11 | (-0.07, 0.28) | 0.65 |
| F_fo/ecf_ | V_b,start_ | 124 | 0.10 | (-0.08, 0.27) | 0.66 |
| V_icf_ | V_r_ | 124 | -0.09 | (-0.26, 0.08) | 0.67 |
| V_fo_ | F_r/u,v,start_ | 120 | 0.09 | (-0.09, 0.26) | 0.74 |
| F_fo/ecf_ | F_b,end_ | 278 | -0.06 | (-0.17, 0.06) | 0.74 |
| F_fo/ecf_ | F_r/u,v,start_ | 120 | 0.08 | (-0.10, 0.26) | 0.74 |
| V_ecf_ | V_b,bm,start_ | 124 | 0.09 | (-0.09, 0.26) | 0.74 |
| V_tbf_ | F_b,end_ | 278 | 0.05 | (-0.07, 0.17) | 0.74 |
| V_tbf_ | F_r/u,v,start_ | 120 | -0.08 | (-0.25, 0.10) | 0.74 |
| V_tbf_ | F_r/u,q,start_ | 123 | -0.08 | (-0.25, 0.10) | 0.74 |
| V_fo_ | F_r/u,q,start_ | 123 | -0.08 | (-0.25, 0.10) | 0.74 |
| V_icf_ | Q_r,start_ | 124 | -0.07 | (-0.24, 0.10) | 0.77 |
| V_fo_ | F_b,end_ | 278 | -0.04 | (-0.16, 0.07) | 0.77 |
| F_fo/ecf_ | F_r/u,q,start_ | 123 | -0.07 | (-0.24, 0.11) | 0.77 |
| V_icf_ | V_b,end_ | 124 | 0.07 | (-0.11, 0.24) | 0.77 |
| V_tbf_ | F_r/u,q,end_ | 122 | -0.07 | (-0.24, 0.11) | 0.77 |
| V_fo_ | V_b,bm,start_ | 124 | 0.06 | (-0.12, 0.23) | 0.78 |
| F_fo/ecf_ | V_b,bm,start_ | 124 | 0.06 | (-0.12, 0.23) | 0.78 |
| V_tbf_ | V_b,bm,start_ | 124 | 0.05 | (-0.12, 0.23) | 0.78 |
| V_tbf_ | V_b,bm,end_ | 124 | 0.06 | (-0.11, 0.24) | 0.78 |
| V_tbf_ | Q_r,start_ | 124 | 0.06 | (-0.12, 0.23) | 0.78 |
| V_icf_ | V_b,bm,end_ | 124 | 0.05 | (-0.13, 0.22) | 0.82 |
| V_tbf_ | F_r/u,v,end_ | 123 | 0.05 | (-0.13, 0.22) | 0.82 |
| V_ecf_ | V_b,bm,end_ | 124 | 0.05 | (-0.13, 0.22) | 0.82 |
| V_ecf_ | F_r/u,q,end_ | 122 | 0.04 | (-0.13, 0.22) | 0.82 |
| V_fo_ | V_b,end_ | 124 | 0.04 | (-0.14, 0.21) | 0.83 |
| F_fo/ecf_ | V_b,end_ | 124 | 0.04 | (-0.14, 0.21) | 0.83 |
| V_icf_ | V_b,start_ | 124 | 0.03 | (-0.14, 0.21) | 0.89 |
| V_tbf_ | Q_r,end_ | 124 | 0.03 | (-0.15, 0.20) | 0.89 |
| V_icf_ | F_r/u,q,start_ | 123 | -0.03 | (-0.20, 0.15) | 0.91 |
| V_icf_ | F_r/u,v,end_ | 123 | -0.02 | (-0.20, 0.15) | 0.94 |
| V_ecf_ | F_r/u,v,start_ | 120 | 0.02 | (-0.16, 0.20) | 0.96 |
| V_icf_ | V_b,bm,start_ | 124 | 0.01 | (-0.17, 0.18) | 0.99 |
| V_fo_ | V_b,bm,end_ | 124 | 0.00 | (-0.17, 0.18) | 0.99 |
| F_fo/ecf_ | V_b,bm,end_ | 124 | 0.00 | (-0.17, 0.18) | 0.99 |
| V_ecf_ | F_b,end_ | 278 | 0.00 | (-0.12, 0.11) | 0.99 |
| V_icf_ | V_u_ | 281 | 0.00 | (-0.11, 0.12) | 0.99 |

Legend to Table S2: Abbreviations: CI, confidence interval; df, degrees of freedom; F, fraction; $\rho_{rm}$, repeated measures correlation coefficient; V, volume. Subscripts: b, blood; bm, post-dialysis body mass; ecf, extracellular fluid; end, corresponding to treatment end; icf, intracellular fluid; isf, interstitial fluid; fo, fluid overload; r, refilling; start, corresponding to treatment start; tbf, total body fluid; u, ultrafiltration.

Table S3: Patient and treatment characteristics at the time of recorded intradialytic hypotensive events

| **Variable** | **Observations** |  |
| --- | --- | --- |
| Time of event, min | 27 | 152 (33, 193) |
| Pre-dialysis fluid overload, L | 27 | 2.4 (1.1, 3.3) |
| Pre-dialysis relative fluid overload, % | 27 | 13.1 (6.0, 17.4) |
| Blood volume, L | 18 | 4.6 (3.7, 4.9) |
| Blood volume normalized to V_ecf_ pre-dialysis, % | 18 | 23.3 (22.4, 30.1) |
| Blood volume, mL/kg post-dialysis body mass | 18 | 49.0 (45.9, 58.0) |
| Blood volume, mL/kg lean tissue mass | 18 | 133.7 (123.3, 179.1) |
| Systolic blood pressure, mmHg | 27 | 85 (73, 88) |
| Relative blood volume at treatment end, % | 27 | 91.1 (87.5, 95.1) |

Legend to Table S3: The data are reported as median (interquartile range) and represent patient and treatment characteristics at the times when intradialytic hypotension occurred based on the Nadir90 and Nadir100 definition (Flythe et al., *J Am Soc Nephrol*, 2015). Symbol: V_ecf_, volume of extracellular fluid.

**Supplemental Figures**

Figure S1: Intradialytic changes of specific blood volume grouped by patient and treatment


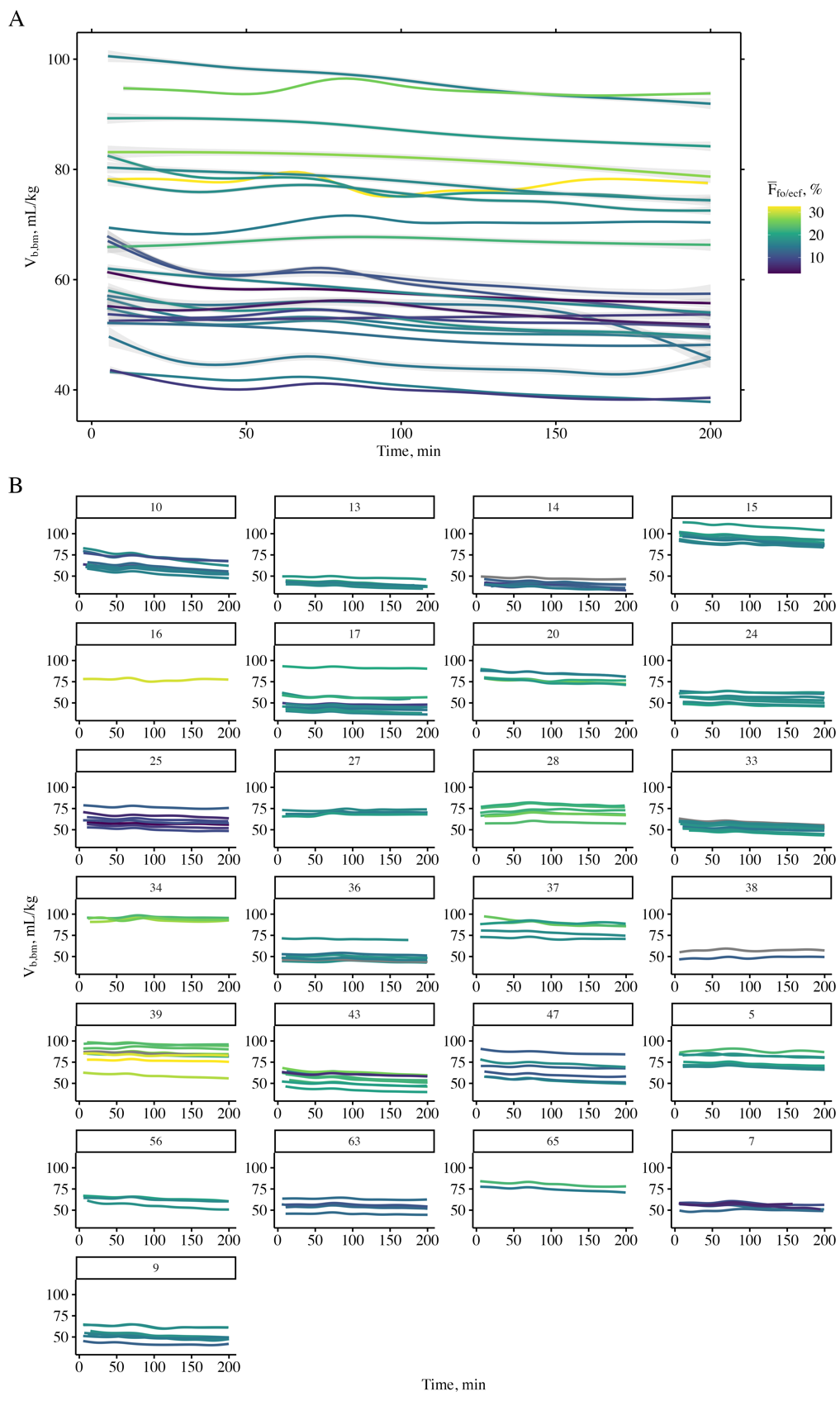


Legend to Figure S1: Panel A shows one GAM-smoothed curve per patient, the color indicating mean relative predialytic fluid overload (F_fo/ecf_) of the respective patient throughout the study. In Panel B, patients are grouped in subpanels (labelled with the patient ID) with each treatment represented by an individual GAM-smoothed curve. The color indicates F_fo/ecf_ of the respective treatment. Symbols: F_fo/ecf_, predialytic fluid overload relative to extracellular fluid volume; V_b,bm_, blood volume normalized to post-dialysis body mass.

Figure S2: Intradialytic changes of ultrafiltration rates grouped by patient and treatment


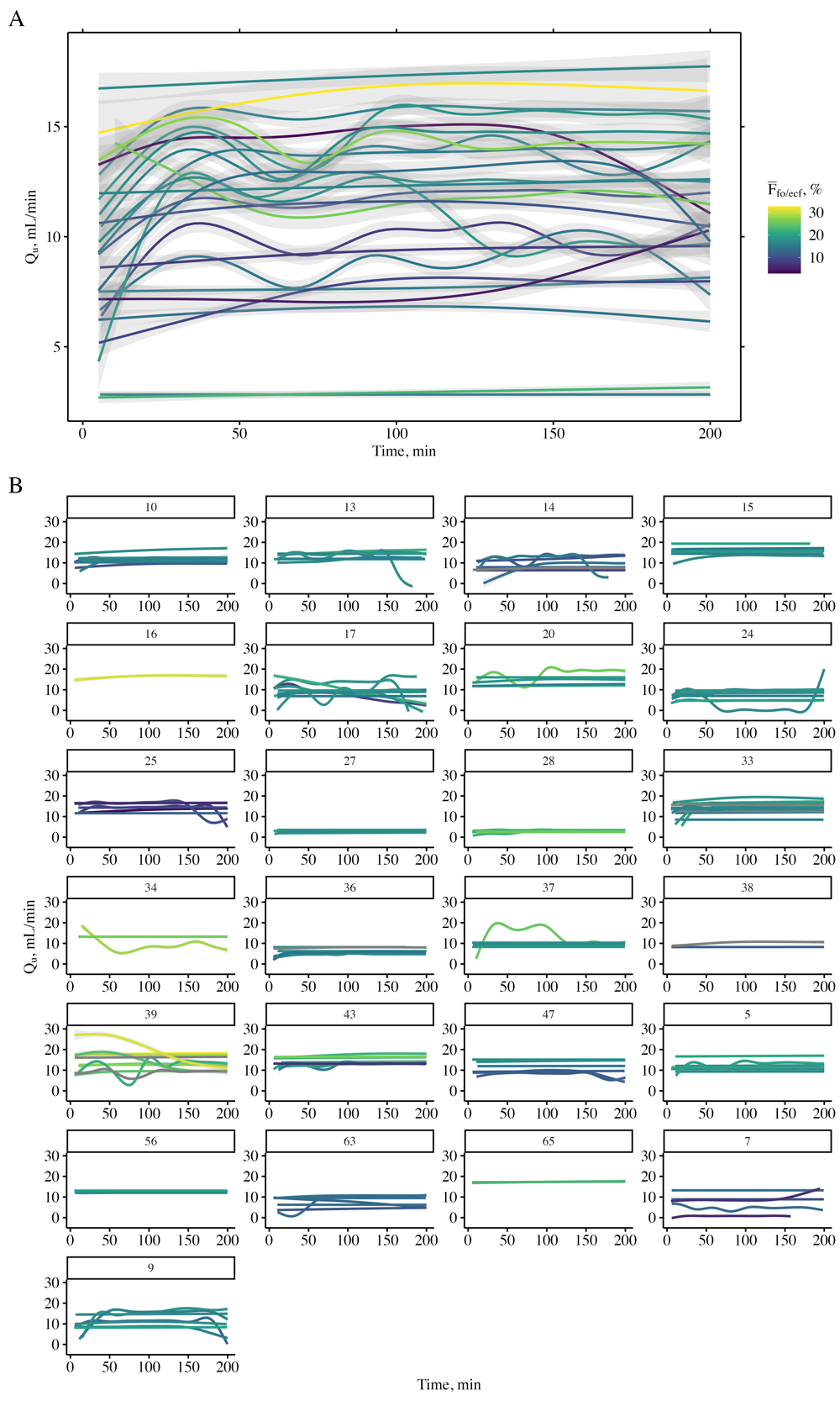


Legend to Figure S2: Panel A shows one GAM-smoothed curve per patient, the color indicating mean relative predialytic fluid overload (F_fo/ecf_) of the respective patient throughout the study. In Panel B, patients are grouped in subpanels (labelled with the patient ID) with each treatment represented by an individual GAM-smoothed curve. The color indicates F_fo/ecf_ of the respective treatment. Symbols: F_fo/ecf_, predialytic fluid overload relative to extracellular fluid volume; V_b,bm_, blood volume normalized to post-dialysis body mass; Q_u_, ultrafiltration rate.

Figure S3: Intradialytic changes of vascular refilling rates grouped by patient and treatment


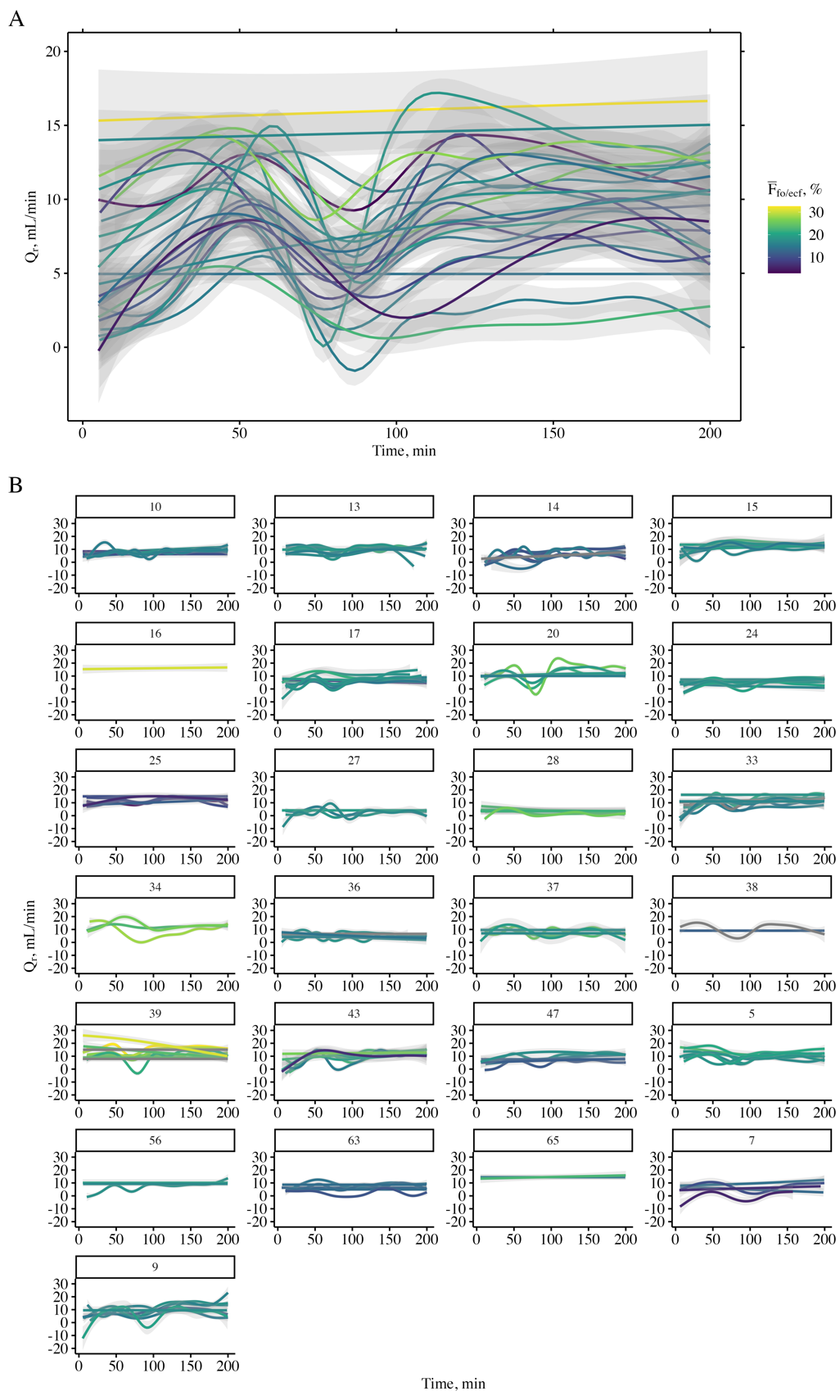


Legend to Figure S3: Panel A shows one GAM-smoothed curve per patient, the color indicating mean relative predialytic fluid overload (F_fo/ecf_) of the respective patient throughout the study. In Panel B, patients are grouped in subpanels (labelled with the patient ID) with each treatment represented by an individual GAM-smoothed curve. The color indicates F_fo/ecf_ of the respective treatment. Symbols: F_fo/ecf_, predialytic fluid overload relative to extracellular fluid volume; V_b,bm_, blood volume normalized to post-dialysis body mass; Q_r_, refilling rate.

Figure S4: Intradialytic changes of the ratio between vascular refilling rate and ultrafiltration rate grouped by patient and treatment


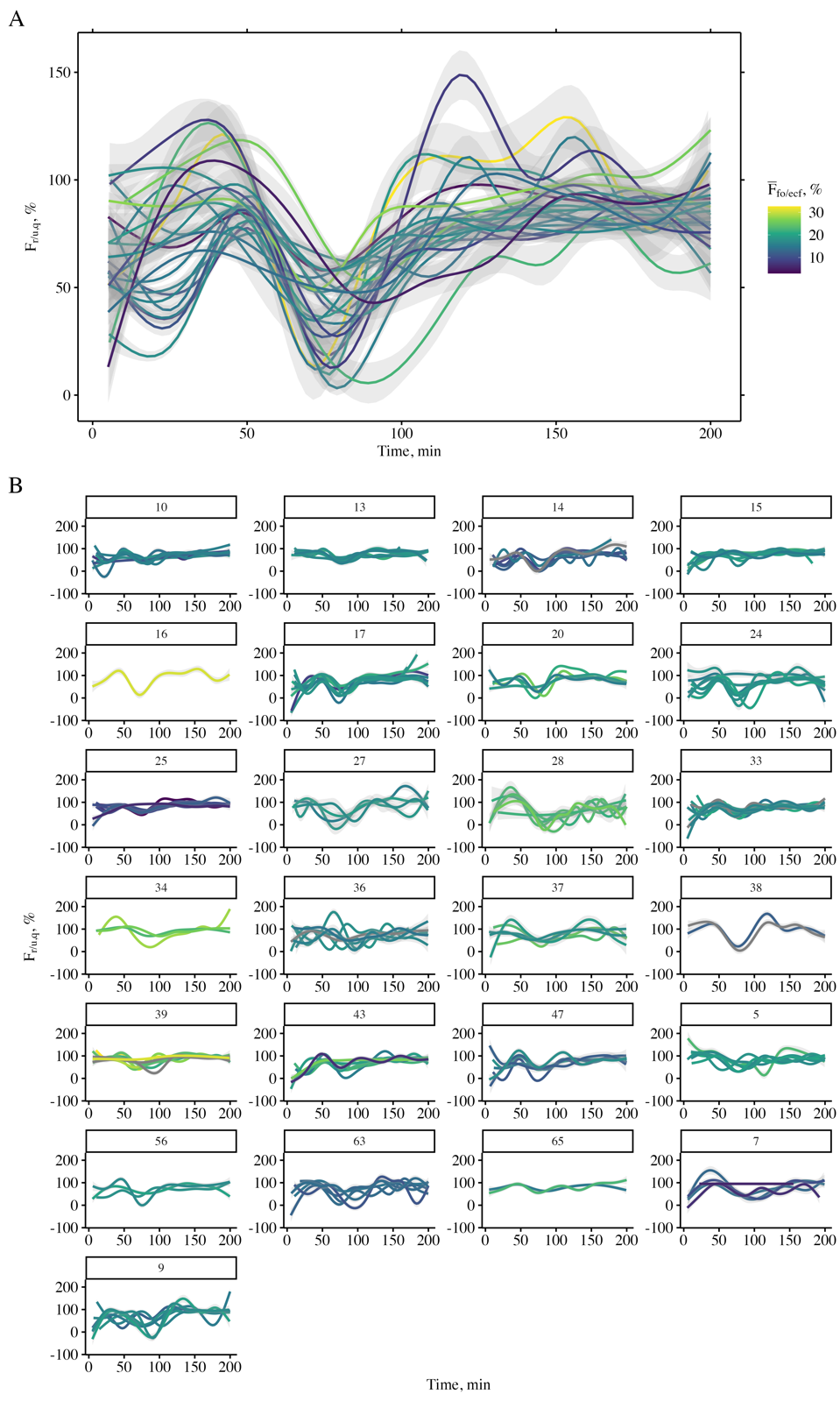


Legend to Figure S4: Panel A shows one GAM-smoothed curve per patient, the color indicating mean relative predialytic fluid overload (F_fo/ecf_) of the respective patient throughout the study. In Panel B, patients are grouped in subpanels (labelled with the patient ID) with each treatment represented by an individual GAM-smoothed curve. The color indicates F_fo/ecf_ of the respective treatment. Symbols: F_fo/ecf_, predialytic fluid overload relative to extracellular fluid volume; V_b,bm_, blood volume normalized to post-dialysis body mass; F_r/u,q_, refilling fraction calculated as the ratio between refilling rate and ultrafiltration rate.

Figure S5: Intradialytic changes of the ratio between vascular refilling volume and ultrafiltration volume grouped by patient and treatment


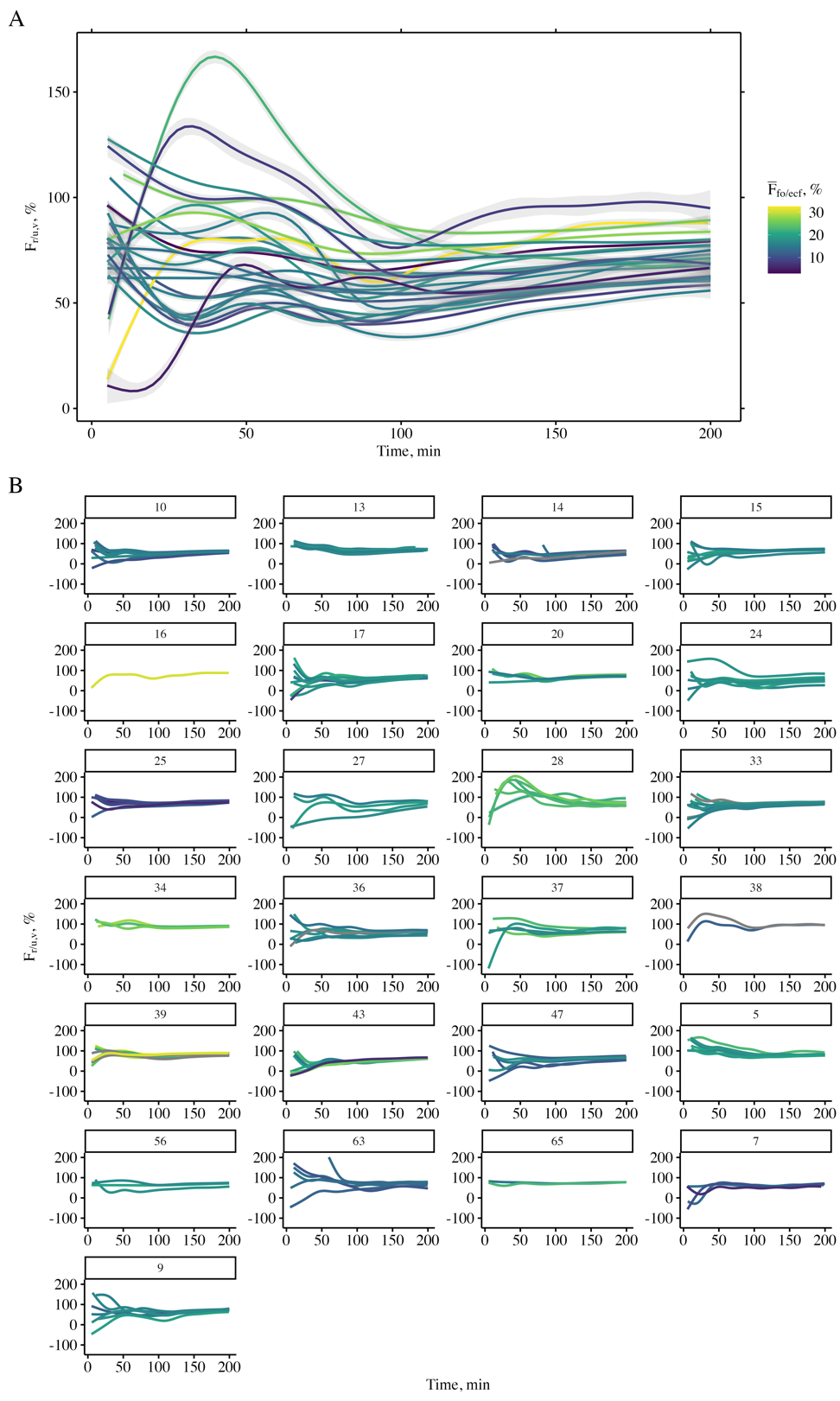


Legend to Figure S5: Panel A shows one GAM-smoothed curve per patient, the color indicating mean relative predialytic fluid overload (F_fo/ecf_) of the respective patient throughout the study. In Panel B, patients are grouped in subpanels (labelled with the patient ID) with each treatment represented by an individual GAM-smoothed curve. The color indicates F_fo/ecf_ of the respective treatment. Symbols: F_fo/ecf_, predialytic fluid overload relative to extracellular fluid volume; V_b,bm_, blood volume normalized to post-dialysis body mass; F_r/u,v_, refilling fraction calculated as the ratio between refilling volume and ultrafiltration volume.

Figure S6: Intradialytic changes of specific blood volume, ultrafiltration and vascular refilling


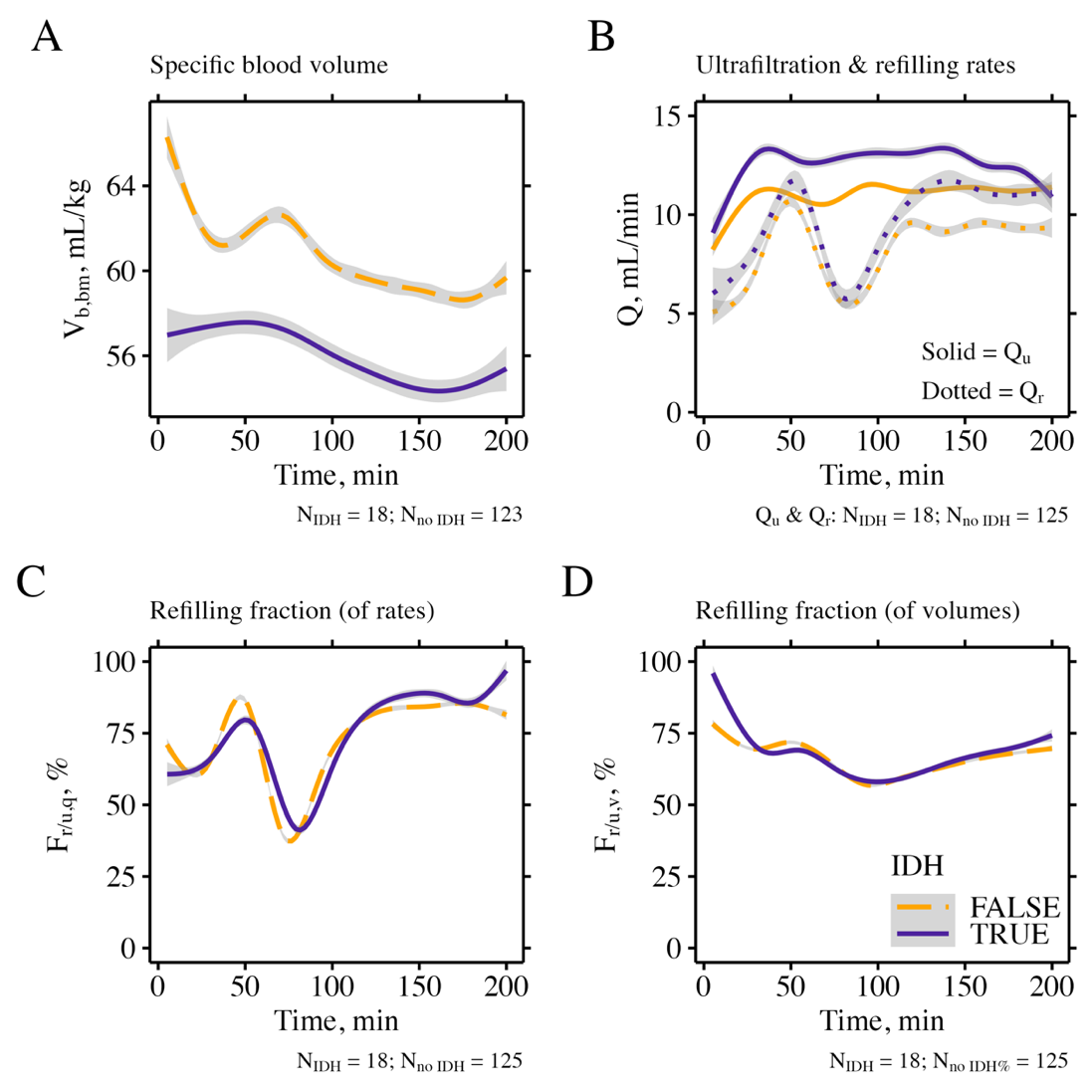


Legend to Figure S6: Patients were stratified based on the presence or absence of intradialytic hypotension during the respective treatment according to the Nadir90/100 classification. Data from all treatments of the respective patient group were fitted by a non-linear smooth function (method ”gam”). Shaded areas delimit the 95% confidence interval. Notice that the disturbance starting around 60 min is caused by the infusion of dialysate to measure absolute blood volume. Time was cut off at 200 min to avoid distortion due to fewer data at later time points. Data on blood volume from the first 5 min of treatment and within 1 min before to 5 min after the dialysate bolus infusion, refilling fractions above 200% and below -100% as well as refilling rates below -50 mL/min were removed from analysis. Abbreviations: F, fraction; IDH, intradialytic hypotension; Q, flow rate; V, volume. Subscripts: b, blood; bm, normalized to post-dialysis body mass; q, refilling fraction calculated from rates (of refilling and ultrafiltration); r, refilling; u, ultrafiltration; v, refilling fraction calculated from volumes (of refilling and ultrafiltration).
